# Meeting report: the 24th Annual Congress of the European College of Sports Science (ECSS) – *3-6 July 2019, Prague, Czech Republic*

**DOI:** 10.1186/s40798-020-00244-z

**Published:** 2020-03-12

**Authors:** Steve McMillan

**Affiliations:** Springer Nature, 5 The Warehouse Way, Northcote, Auckland, New Zealand

## Introduction

The 2019 European College of Sports Science (ECSS) congress was held in Prague, under the patronage of Charles University. There were approximately 2800 delegates in attendance, from around 80 countries. The programme consisted of four plenaries; six industry-sponsored satellites/workshops; 39 invited-speaker sessions; and around 136 other oral presentation sessions, as well as poster sessions involving conventional posters and electronic posters. Below, I have summarised three presentations that I considered to be highlights:

### Dr. Aki Salo, in the opening plenary, on optimising sprint performance

If you are a sprinter wanting to reach the finish line sooner (a common goal for most sprinters), you can take quicker steps; you can lengthen your steps (to cover more ground with each step); or you can try to do both.

Of course, in the short term at least, it is nigh on impossible to achieve both: longer strides take longer to perform and, on the flipside, ramping up one’s step rate inevitably causes one to take shorter steps, which is counter-productive. Given this inevitable trade-off, one has to make a choice: to either increase step frequency or stride length; so, which should one choose?

Cross-sectional data [1] indicate that the average athlete gets a greater payoff from lengthening their stride. On average, there is a stronger correlation between improvements in stride length and improvements in velocity than there is between improvements in step rate and velocity. However, in the first of the four plenaries presented at ECSS, Dr. Aki Salo (Director of the Research Institute for Olympic Sports, in Finland) showed that there really is no `average athlete’, so these cross-sectional data are not that helpful [2].

Competition data from a group of elite male sprinters showed that each athlete was pitched at a different point along a continuum from relying heavily on improvements in stride length to achieve peak performance to relying heavily on step frequency to achieve peak performance [3]. As an extreme example, Salo showed longitudinal data from a 60-m indoor world champion [4]; for whom, improvements in step frequency showed a strong linear correlation with improvements in sprint speed (R^2^ = 0.79), with the athlete giving his best performances at step frequencies in excess of 5 Hz! Attempts to increase step length, at certain periods over four months of training, were not correlated with improvements in speed at all (R^2^ = 0.04) … if anything, there was a tendency for increases in stride length to worsen the athlete’s performance.

Dr. Salo explained that, while sprinting requires both horizontal and vertical force production, better sprinters are more capable of orienting their force production in a horizontal direction. Traditionally, when analysing performance, researchers have been limited to focusing on the assessment of discrete variables on the vertical and/or horizontal (anteroposterior) force curves, such as maximum vertical force, maximum horizontal force, maximum braking force, etc. However, using an amazing indoor track in Kanoya, Japan, that has force-plates embedded the entire way along (54 in total), and using a novel approach to analyse complete ground reaction force waveforms rather than discrete kinetic variables, Salo, together with Steffi Colyer and Ryu Nagahara, analysed anteroposterior force waveforms over multiple steps in maximal-effort sprints performed by elite sprinters [5]. The researchers found that, while maximum force production in the initial few steps was associated with better performance, from about the seventh step onwards, maximum force production was no longer associated with better performance. As the sprint progressed, force production in each step waveform became progressively more eccentric; as maximum velocity was approached (in the second half of the sprint) the more successful sprinters were those who were better at attenuating the eccentric braking forces.

When comparing the sprint force waveforms of experienced sprinters to those of soccer players, Salo and colleagues showed that sprinters produced greater horizontal force around the middle of the stance phase (from ~ 30% of the way through the stance phase to ~ 80% of the way through the stance phase), as well as greater horizontal force at the very end of the stance phase [6]. Dr. Salo therefore advised that, rather than focusing on improving maximal force production, for instance, more focus should be placed on improving force production throughout the middle part of the stance phase. The question then remains, `*how does one improve force production during this middle part of the stance phase?*’

Dr. Salo concluded his presentation by noting that a key limitation to the research conducted to date is that it is based on observations of how athletes actually perform, rather than trying to determine what *optimal* performance looks like (one can try to mimic top athletes, but even top athletes may not be performing optimally) [2]. Computer modelling can be used to try to determine optimal performance, but modelling analyses have traditionally been limited to considering the effects of changing one variable at a time. Only recently have models become sophisticated enough to account for the ramifications changing one particular variable has in terms of changing other variables. Salo indicated that we are probably a few years away from being able to use models effectively to select the specific aspects of an individual athlete’s training that should be targeted (for instance, what muscle groups should be strengthened) in order to improve that particular individual’s performance. There is then also the question of whether targeted training can actually improve one’s performance, for which, longitudinal studies are required.

### The young investigator award winner, Yves-Alain Kuhn, on balance training and brain adaptations in the elderly

The Young Investigator Award was won by Mr. Yves-Alain Kuhn, of the University of Fribourg, for his presentation of an elegant study investigating the neural mechanisms through which balance training can help reduce fall risk in the elderly [7].

Postural control deteriorates with age, due to declines in peripheral nerve fibres and other sensory structures, as well as declines in total brain volume, and the number and size of skeletal muscle fibres. These structural changes are accompanied by (perhaps compensated by) greater, and more widespread, activation of the cortical areas of the brain [8].

Mr. Kuhn explained that, basically, with aging, postural control goes from being an automated process, employing lower levels of the brain, to being a more attention-demanding activity, reflected by greater, and more widespread, cortical activation [7].

Interneurons within the cortex can either be classified as excitatory or inhibitory. Overall increases in cortical activation result from the up-regulation of excitatory interneurons and/or the down-regulation of inhibitory interneurons. Using a particular technique of transcranial magnetic stimulation (TMS) that involves pairing two magnetic stimuli – milliseconds apart – over the primary motor cortex, one can assess the activity of GABA-A inhibitory interneurons within the motor cortex; this activity is referred to as short-interval intracortical inhibition, or `SICI’ [9].

Kuhn and colleagues showed that, when performing a standing balance task, participants aged 66–81 years old expressed a down-regulation of SICI (approximately 20% less SICI) compared with participants aged 21–34 years. With balance training, however, this age-associated cortical disinhibition could be reversed, such that the levels of SICI in the older participants were brought closer to those of their younger counterparts [7].

Kuhn et al. randomised 33 elderly participants to either a balance training group (who underwent balance training for one hour, two times per week) or a control group. After 6 months of training, the balance training group showed significant improvements in balance performance compared with controls. The balance training group also showed significant increases in SICI when balancing, whereas the control group showed no changes in levels of SICI. There was a significant correlation between balance performance and SICI at the six-month endpoint.

At 12 months, after six months of de-training, while the effects of training wore off to some extent, the balance training group still showed better balance performance and greater levels of SICI compared with controls.

### Prof. Ann McKee, in the third plenary, on the devastating effects of chronic traumatic encephalopathy 

In the third of four plenaries, Prof. Ann McKee, Director of Boston University’s Chronic Traumatic Encephalopathy (CTE) Center, presented a slide showing a cross-section of the brain of Aaron Hernandez, a US National Football League (NFL) player who played for New England Patriots before being arrested for murder and, four years later, committing suicide, at the age of 27 [10].

Prof. McKee did not open her presentation with this slide, nor was this image intended to represent the be-all-and-end-all of Prof. McKee’s presentation, which covered 12 years of research since launching the world’s first, and now largest, brain bank for cases of CTE. However, like a graphical health warning on a cigarette packet, the image of Hernandez’ brain poignantly demonstrated the horrific effects that repetitive head impacts can have.

Prof. McKee explained how CTE is definitively diagnosed by considering brain slices under the microscope; the pathognomonic signs of CTE are the accumulation of tau protein and neurofibrillar tangles circling small blood vessels, and the irregular, patchy accumulation of lesions deep in the brain’s sulci. Early stages of the disease involve only a few isolated spots, usually in the frontal lobe or perhaps the temporal lobe. As the disease progresses, these spots spread: they become enlarged, and more numerous, distributed widely throughout the frontal, parietal and temporal lobes; then into the medial temporal lobe, hippocampus, amygdala and entorhinal cortex. By the end-stage (Stage IV), the disease is very widespread, involving almost all parts of the brain, as well as the spinal cord and brain stem.

In the case of Aaron Hernandez, one did not need a microscope to conclude that the brain was unhealthy. The severity of Hernandez’s disease was such that a cross-section of the whole brain showed enormous ventricular dilatation relative to the brain of a deceased aged-matched control, as well as atrophy of the fornix, and striking perforations of the septum pellucidum. Under the microscope, tau pathology could be seen throughout the brain, particularly in the frontal cortex where half the area was riddled with lesions.

Boston University’s CTE brain bank is currently growing at a rate of 6 or 7 cases a day; it contains over 750 brains from donors participating in a wide range of sports including American football, rugby, boxing, wrestling, ice hockey, soccer, baseball, mixed martial arts, bull riding and motocross.

To date, CTE has only been diagnosed in cases exposed to physical trauma. In a 2015 study of more than 1700 adult male brains from the Mayo Clinic Brain Bank [11], CTE pathology was identified in 21 of 66 contact-sport athletes (32%), while there were no cases of CTE among 198 brains that had come from cases with no history of trauma or contact-sport exposure. There were also no cases of CTE among 33 brains that each had a history of only a single traumatic event, indicating that CTE results from exposure to repetitive head impact.

The tissue strain that occurs during the kind of trauma that contact-sport participants are exposed to, explains the placement of the tau pathology [10]. The linear and rotational accelerations that the brain endures upon impact result in the greatest stress forces being placed at the interface of the blood vessels and the brain parenchyma, two tissues of different viscoelastic properties, as well as in the depths of the brain’s crevasses (sulci).

It seems that, at least in some people, once it starts, CTE continues to spread in the absence of further trauma. The mean age for Stage II disease cases is 44 years, whereas the mean ages for the more severe stages (Stages III and IV) are 56 and 77 years, respectively. Thus, cases are showing disease progression at ages that are typically beyond sport retirement.

As devastating as CTE might be, athletes are unlikely to be deterred from participating in their chosen sport if they believe that the risks to them are low. An often-cited limitation of brain bank research is that disease prevalence cannot be accurately determined, because of the inherent selection bias that occurs when one is reliant upon voluntary brain donations. McKee, however, presented some published research involving estimates.

A case series of brains from 202 American football players, from all levels of play, published in *JAMA*, found that 87% were diagnosed with CTE [12]. Among those who played in the NFL, 99% (110 of 111) were diagnosed with CTE. Over the period that this study was conducted, 1300 other former NFL players passed away. If one was to assume, conservatively, that all of the former NFL players whose brains were not donated to the brain bank were negative for CTE, the total estimated prevalence of CTE among these former NFL players would be one in ten.

This estimate was supported in a subsequent analysis conducted by Zachary Binney and Kathleen Bachynski, published in *Neurology* [13]. These researchers showed (similarly) that, if 100% of the cases of CTE diagnosed post-mortem in former NFL players were being captured by the CTE brain bank (i.e. there were no other cases of CTE among deceased NFL players over the same time period), the prevalence would be 10%. Of course, since the brain bank relies on donations, it is unlikely to be capturing 100% of cases. If only some of the cases of CTE were being donated to the brain bank (e.g. 30% or 50%), the estimated prevalence would be much higher (if 50% of affected brains were donated, the prevalence among former NFL players would be approximately 19%). The researchers showed that the relationship between the probability of donation among cases of CTE and the estimated prevalence of CTE was exponential - to the extent that - if, theoretically, only 10% of the deceased former NFL players with CTE had donated their brains to the brain bank, the overall prevalence among this population would be close to 100%.

In the same way that one cannot prove that smoking causes lung cancer, one cannot prove that trauma causes CTE; however, dose-response relationships have been shown in American football players: between years of play and higher levels of tau protein, higher levels of inflammation, and higher levels of dementia [12, 14, 15]. Also, in living high school and collegiate football players, the estimated number of cumulative head impacts has been found to be associated with late-life depression, apathy, executive dysfunction and cognitive impairment [16].

Prof. McKee advised that, as it is the cumulative exposure to sub-concussive head impacts that confers risk for CTE, raising the age that children play contact sports would reduce the risk [10]. While research has yet to determine if there is a critical exposure threshold for CTE, if children were to simply begin contact sport at 15 years of age, rather than five, they would have a decade less exposure.

There’s currently no way of diagnosing CTE in the living, and there are no known treatments. The monitoring of plasma tau levels, and tau PET scans, have shown some promise as diagnostic methods, and research is ongoing to determine inflammatory cytokines that might be unique to CTE. McKee points out that there are dozens of treatments currently available that might be effective against CTE, but until there is a way to detect CTE in the living, one cannot monitor treatment efficacy.
